# The Use of Nanotrap Particles in the Enhanced Detection of Rift Valley Fever Virus Nucleoprotein

**DOI:** 10.1371/journal.pone.0128215

**Published:** 2015-05-28

**Authors:** Nazly Shafagati, Lindsay Lundberg, Alan Baer, Alexis Patanarut, Katherine Fite, Benjamin Lepene, Kylene Kehn-Hall

**Affiliations:** 1 National Center for Biodefense and Infectious Diseases, School of Systems Biology, George Mason University, Manassas, Virginia, United States of America; 2 Ceres Nanoscience, Manassas, Virginia, United States of America; The University of Texas Medical Branch, UNITED STATES

## Abstract

**Background:**

Rift Valley fever virus (RVFV) is a highly pathogenic arthropod-borne virus that has a detrimental effect on both livestock and human populations. While there are several diagnostic methodologies available for RVFV detection, many are not sensitive enough to diagnose early infections. Furthermore, detection may be hindered by high abundant proteins such as albumin. Previous findings have shown that Nanotrap particles can be used to significantly enhance detection of various small analytes of low abundance. We have expanded upon this repertoire to show that this simple and efficient sample preparation technology can drastically improve the detection of the RVFV nucleoprotein (NP), the most abundant and widely used viral protein for RVFV diagnostics.

**Results:**

After screening multiple Nanotrap particle architectures, we found that one particle, NT45, was optimal for RVFV NP capture, as demonstrated by western blotting. NT45 significantly enhanced detection of the NP at levels undetectable without the technology. Importantly, we demonstrated that Nanotrap particles are capable of concentrating NP in a number of matrices, including infected cell lysates, viral supernatants, and animal sera. Specifically, NT45 enhanced detection of NP at various viral titers, multiplicity of infections, and time points. Our most dramatic results were observed in spiked serum samples, where high abundance serum proteins hindered detection of NP without Nanotrap particles. Nanotrap particles allowed for sample cleanup and subsequent detection of RVFV NP. Finally, we demonstrated that incubation of our samples with Nanotrap particles protects the NP from degradation over extended periods of time (up to 120 hours) and at elevated temperatures (at 37ºC).

**Conclusion:**

This study demonstrates that Nanotrap particles are capable of drastically lowering the limit of detection for RVFV NP by capturing, concentrating, and preserving RVFV NP in clinically relevant matrices. These studies can be extended to a wide range of pathogens and their analytes of diagnostic interest.

## Introduction

Rift Valley fever virus (RVFV) is a zoonotic virus belonging to the genus *Phlebovirus* within the family *Bunyaviridae*. This highly pathogenic virus is transmitted to a wide range of vertebrate hosts, primarily livestock and humans, via mosquitoes and other arthropod vectors. Humans may become infected through contact with infected bodily fluids or tissues, mosquito bites, or aerosolization [1]. While symptoms in humans are usually mild and resemble a flu-like illness, a small percentage of patients (~2%) may develop serious disease, which includes retinal lesions, meningoencephalitis, hepatitis, severe hemorrhagic fever, coma and death. In recent years, there has been an increased rate of mortality in humans (>30%) [2,3]. Mortality rates in livestock are more dramatic; as high as 30% in adult livestock and up to 100% in newborns and the young [4].

RVFV can be detected by virus isolation, enzyme-linked immunosorbent assay (ELISA), or molecular techniques, such as reverse-transcription PCR (RT-PCR) [5]. However each of these methodologies have their caveats. For example, virus isolation can be both costly and time-consuming. Molecular based assays such as RT-PCR, quantitative RT-PCR (qRT-PCR) using the TaqMan probe technology, and real-time reverse-transcription loop-mediated isothermal amplification assays (RT-LAMP) while highly specific and sensitive are time-consuming and require specialized laboratory equipment and trained personal [5]. The nucleoprotein (NP) of RVFV is the most abundant viral protein and is often used in diagnostic testing by ELISA. ELISAs are affordable and generally regarded as specific, but the detection limit is significantly lower than qRT-PCR assays [5]. Furthermore, NP was observed to serologically cross-react during testing of sera obtained from US and Canadian livestock [6]. Thus, there is a need for a sample preparation method that will eliminate highly abundant interfering proteins and concentrate viral antigens. Nanotrap particles are a novel sample preparation technology that can address these issues.

Nanotrap particles are hydrogel particles of various sizes (from 300 nanometers up to 3000 nanometers). These hydrogel particles contain a core made of cross-linked polymeric networks that consist of N-isopropylacrylamide (NIPAm) and co-monomers such as acrylic acid (AAc), allylamine (AA), and N,N′-methylenebisacrylamide (BIS). Nanotrap particles are functionalized with charge-based affinity baits via copolymerization and covalent binding to the shell. The baits serve to further capture, concentrate, and trap targets proteins. The promiscuity of these particles can further be decreased by encapsulating the particles within an outer cross-linked poly(p)-NIPAm shell that may be inert or contain vinyl sulfonic acid (VSA). This shell can be modified for the inclusion or exclusion of high abundant large molecules such as albumin by increasing or decreasing the size of the pores, which are generated by cross-linking [7–9].

Nanotrap particles increase sensitivity by performing three important functions in one step: molecular size sieving, target analyte affinity sequestration, and protection of the captured analyte from degradation. Several studies conducted by Luchini *et al*. demonstrated the capture and enrichment of small molecules spiked in whole blood, serum, and other complex solutions [7,9]. Recent studies have utilized Nanotrap particles in viral and bacterial infections. Nanotrap particles have been used to increase the sensitivity of Lyme disease detection by capturing the antigen shed by *Borrelia burgdorferi*, the bacteria responsible for the disease, in urine [10]. Another recent paper published by our group utilized these particles as an important sample preparation tool in RVFV detection, where particles allowed for capture, enrichment, and protection of the RVFV virions spiked in serum samples [11]. A 2014 paper by Jaworski *et al*. demonstrated that Nanotrap particles can be utilized to capture both HIV-1 virions as well as various viral proteins such as Tat and Nef in clinically relevant matrices [12]. Nanotrap particles have also been shown to enrich for virions of both RNA and DNA viruses that cause respiratory illnesses, including human coronavirus (HCoV), adenovirus, and influenza A [13]. These studies together have demonstrated that Nanotrap particles can improve sensitivity more than 100-fold over existing methods.

Here we have utilized Nanotrap particles to capture and enrich the RVFV NP from cell lysates, viral supernatants, and animal sera. The enrichment of NP allows for enhanced detection in various downstream assays that require small sample volumes, such as mass spectrometry, ELISA, and western blotting. Our studies demonstrate that the Nanotrap particles are not only capable of enriching NP, but protecting the protein from degradation over time and at increased temperatures. This will allow for easy transfer of samples from the field to laboratories, where incorporation into various downstream diagnostic or experimental assays may be performed.

## Materials and Methods

### Nanotrap particles

NIPAm/AA Nanotrap particles were provided by Ceres Nanoscience, Manassas, VA.

### Cell culture

Vero cells (African green monkey kidney epithelial cells) were grown in Dulbecco's Modified Eagle Medium (DMEM) supplemented with 10% FBS, 1% penicillin/streptomycin, and 1% glutamax (DMEM+++) in a humidified environment containing 5% CO_2_ at 37°C.

### Rift Valley fever virus strain MP12

The experiments described in this paper used a live attenuated vaccine (MP12) derived from the RVFV ZH548 strain that had been isolated in 1977 from a patient with uncomplicated RVFV. The virus was generated by 12 serial passages in MRC5 cells, inducing 25 nucleotide changes across the viral genome [14].

### Animal sera

Goat and sheep sera were obtained from Innovative Research.

### Purified and his-tagged nucleoprotein

The untagged purified NP was obtained from BEI Resources. His-tagged NP was produced in house or obtained from Immune Technology. His-NP plasmid was obtained from Dr. Stuart Nichol (Centers for Disease Control). An agar plate containing Ampicillin was streaked with *E*. *coli* stock and incubated at 37°C overnight. A single colony was isolated, placed in 10 ml of LB broth (containing Ampicillin, MgSO_4_, and dextrose) and shaken overnight at 37°C. Five milliliters (ml) of the overnight culture was transferred into 100 ml of LB broth and shaken at 37°C until an OD_600_ reading of 0.6 was achieved. The expression of His-protein was then induced by adding IPTG at a final concentration of 1.0 mM. The sample was shaken for an additional 4 hours followed by centrifugation at 4,000 rpm for 20 minutes at 4°C. The bacterial pellet was resuspended in 10 ml of lysis buffer (containing 50 mM NaH_2_PO_4_, 300 mM NaCl, 10 mM imidazole, pH 8.0) and lysozyme at a final concentration of 1mg/ml. The sample was incubated on ice for 30 minutes before the cells were lysed by vortexing and sonication. After centrifugation (10,000 rpm for 20 minutes at 4°C), the lysed *E*. *coli* supernatant was transferred to a new tube where a volume of 450 μl of Nickel beads (30% slurry) were added and the sample was rotated for one hour at 4°C. The beads were then washed three times with 5 ml of Wash Buffer (containing 50 mM NaH_2_PO_4_, 300 mM NaCl, 20 mM imidazole, pH 8.0) and the His-tagged protein was eluted with 500 μl of Elution Buffer (containing 50 mM NaH_2_PO_4_, 300 mM NaCl, 250 mM imidazole, pH 8.0). Purity and relative protein concentration were determined by analyzing 10 μl of the His-NP along with known BSA concentrations by SDS-PAGE and commassie staining.

### Standard Nanotrap particle incubation

One hundred μL to 1 mL of sample were incubated with 75–100 μL of Nanotrap particles for 30 minutes at room temperature. Samples were centrifuged at 10,000–14,000 rpm for 10 minutes and supernatants discarded. For serum samples, pellets were washed one time with 0.25 M sodium thiocyanate and two times with RNase- and DNase-free water. The volume of sodium thiocyanate and water washes were equivalent to the volume of original sample used.

### Viral infections

RVFV MP12 used in these experiments was propagated by infecting Vero cells at 80–90% confluency at an MOI of 0.1, 1, 3, or 10 in DMEM+++. Cell culture medium was collected at various timepoints (8, 16, 24, or 48 hours post-infection). Cells were infected by overlaying a 400 μl suspension of viral media on cells plated in a 6-well plate and incubated for one hour at 37°C in a humidified environment containing 5% CO_2_. Following incubation, the viral media was removed and the cells were washed with phosphate buffered saline (PBS) without Mg and Ca and replaced with 2 ml of DMEM+++. Cells were maintained at 37°C at 5% CO_2_ until the appropriate collection time. After collection, viral supernatants were centrifuged at 10,000 rpm for 10 minutes to pellet the cellular debris and then filtered using a 0.22 μM filter.

### Molecular weight separation of viral supernatants

Vivaspin 20 centrifugal concentrators with a 300,000 molecular weight cut-off (MWCO) were used to separate viral supernatants. Five milliliters of viral supernatant were added to the concentrators and centrifuged for 7 minutes at 1400 rpm until approximately 500 μl volume remained on the top portion of the concentrator. Both top and bottom portions, as well as control samples that were not run through the concentrator, were saved for downstream analysis. Plaque assays were performed on control, top, and bottom samples using Vero cells in 12 well plates as previously described by Baer et al. [15]. The band analysis tools (volume box option) of Quantity One 1-D analysis software (Bio-Rad) were used to determine the background-subtracted density of the NP bands in the western blots. The percent of total NP was determined by dividing the top or bottom portion's band density (as determined by western blotting and densitometry analysis) by the total NP band density (top and bottom portions added together) and multiplying by 100.

### Western blot analysis

Nanotrap pellets were resuspended in 20 μL of lysis buffer [containing 1:1 mixture of T-PER reagent (Pierce, IL), 2× Tris-glycine SDS sample buffer (Novex, Invitrogen), 33 mM DTT, and protease and phosphatase inhibitor cocktail (1× Halt cocktail, Pierce)]. No Nanotrap control samples were resuspended in 10 μL of lysis buffer. All samples were boiled for 10 min. The Nanotrap samples were then centrifuged at 14,000 rpm, the supernatant was saved and centrifuged for a second time. The supernatants were separated on NuPAGE 4–12% Bis-Tris gels (Invitrogen) and transferred to PVDF membranes using the overnight wet transfer methodology at 80 mA at 4°C. The membranes were blocked with 3% bovine serum albumin (BSA) in PBS +0.1% Tween-20 (PBS-T) for 1 hour at room temperature. The primary antibodies were diluted in 3% BSA in PBS-T at a 1:1000 dilution for anti-His antibody (Cell Signaling) and at a 1:120 dilution for EC13 and EC22 antibodies (Institute for Infectious Animal Diseases, College Station, TX), and incubated overnight at room temperature. The EC13 and EC22 primary antibodies were reused for several experiments without the addition of extra antibody. The membranes were then washed 3 times with PBS-T and incubated with secondary HRP-coupled goat anti-rabbit (for anti-His antibody) or anti-mouse (for EC13 antibody) antibody diluted 1:1,000 in 3% BSA for 2 hours and then washed 4 times with PBS-T for 5 minutes. The western blots were visualized by chemiluminescence using SuperSignal West Femto Maximum Sensitivity Substrate kit (ThermoScientific) and a Bio-Rad Molecular Imager ChemiDoc XRS system (Bio-Rad). The band analysis tools (volume box option) of Quantity One 1-D analysis software (Bio-Rad) were used to determine the background-subtracted density of the bands in the western blots. For each sample, the averages of two band densities were taken. Fold enrichment was determined by dividing the (+)NT samples by the (-)NT control samples.

### Coomassie gel

NP was eluted from the Nanotrap pellets as described above and loaded on a NuPAGE 4–12% Bis-Tris gel (Invitrogen). After electrophoresis, the gel was covered with Coomassie stain (0.5 g Coomassie Blue R, 800 ml methanol, 140 ml acetic acid, brought up to 2 L with diH_2_O), microwaved until gently boiling, then rocked for 1 hour at room temperature. The Coomassie stain was removed and fresh destaining solution (800 ml methanol, 140 ml acetic acid, brought up to 2 L with diH_2_O) was added until excess stain was removed.

### Cytoplasmic extracts

Vero cells at 8E+06 cells/well were infected with RVFV MP12 at an MOI of 3 and collected after 24 hours. Lysates were resupended in 200 μl of lysis buffer (containing 50mM Tris-HCl, 0.5% Triton X-100, 137.5mM NaCl, 10% glycerol, 1mM NaVaO_4_, 50mM NaF, 10mM sodium pyrophosphate, 5 mM EDTA, and a protease inhibitor cocktail tablet) and incubated on ice for 15 minutes. The sample was then centrifuged at 3,000 rpm for 5 minutes at 4^°^C and the supernatant was saved for further analysis.

### Control and-NT samples

Control samples used throughout the paper consisted of 10 μl of sample (containing no Nanotrap particles) added to 10ul of blue lysis buffer. Samples not incubated with Nanotrap particles (-NT samples) consisted of 10 μl of the sample incubated at room temperature for 30 minutes (in parallel with Nanotrap particle-incubated samples) and then added to 10ul of blue lysis buffer. Both the control and-NT samples were boiled and centrifuged in parallel with +NT samples.

## Results

### Non-virion associated NP can be detected in viral supernatants

**Fig 1:**
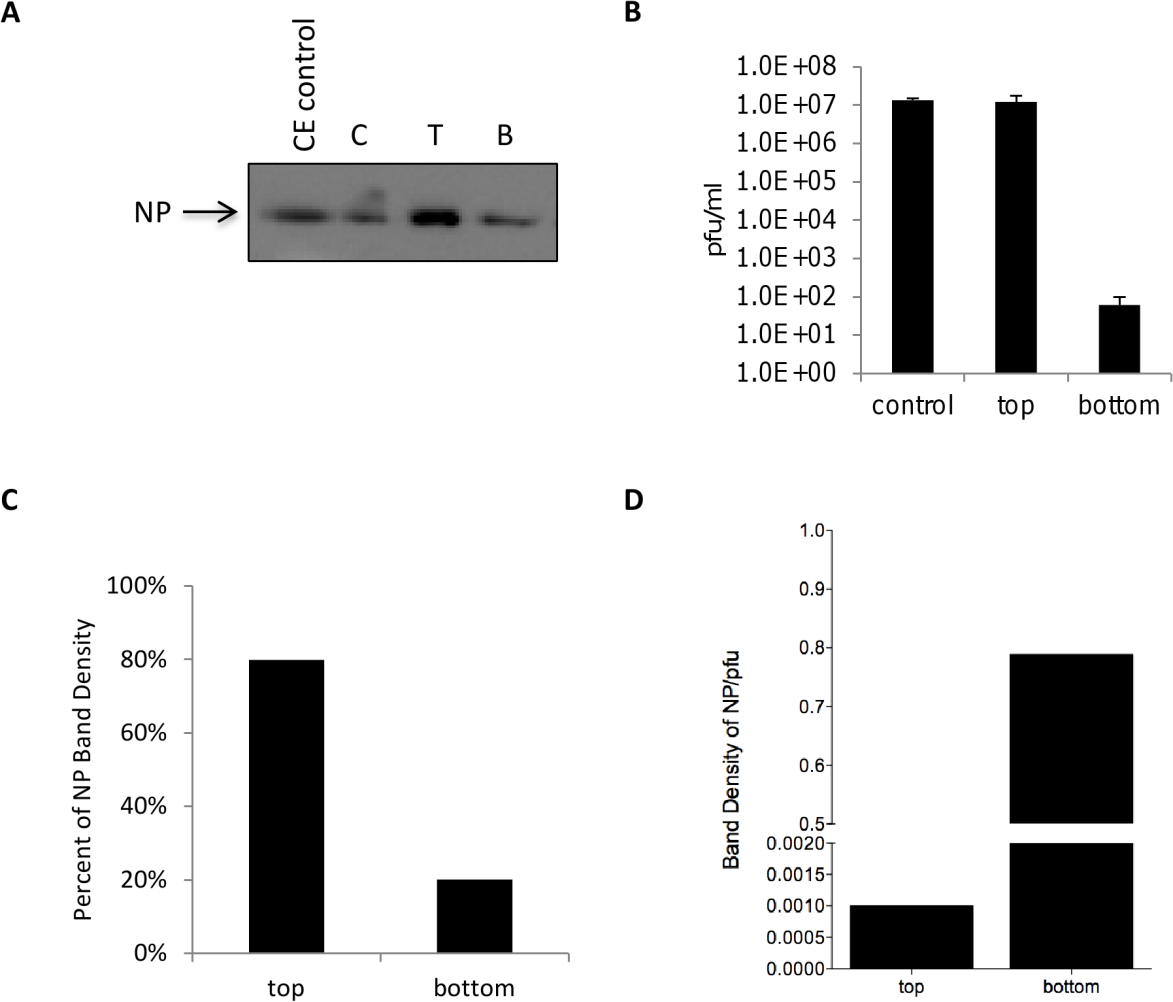
Non-virion associated NP can be detected in viral supernatants. Vivaspin 20 centrifugal concentrators with a 300,000 Da MWCO were used to filter viral supernatants harvested from Vero cells infected at an MOI 1 with MP12. Five milliliters of viral supernatant was added to the concentrators and centrifuged at 1400 rpm for 7 minutes until approximately 500 μl remained on the top portion of the concentrator. A) The top (T) and bottom (B) fractions, as well as control sample (C) containing the original sample before processing were analyzed by western blot using antibodies against NP (EC22 antibody). C, T, and B lysates were undiluted (neat). Cytoplasmic extract (CE) control is 7.7 μg/ml of RVFV infected Vero cell lysates. B) Plaque assays were performed with both fractions and the control sample. C) Densitometry analysis was performed to determine the NP band densities in the T and B fractions. The percent of total NP was determined by dividing the top or bottom portion's band density by the total NP band density (top and bottom portions added together) and multiplying by 100. D) NP band density per pfu values were calculated by dividing the NP band densities by the total pfu values.

Nucleoprotein (NP) is the most abundant viral protein of RVFV. It has been previously speculated that NP of RVFV and other viruses can be released from host cells in the absence of other proteins. A paper published by Liu *et al*. demonstrated that a substantial amount of NP could be purified from the media of infected Sf9 cells, suggesting that this protein may be released independently of the other viral proteins [16]. Therefore, RVFV NP can be utilized as an important biomarker in detecting RVFV infections. Our first set of experiments sought to confirm the presence of non-virion associated NP in viral supernatants. Vero cells were infected with RVFV MP12 (MOI 1) and media supernatants collected when 80–90% CPE was observed (48 hours post infection). Supernatants were separated using a centrifugal concentrator with a molecular weight cutoff (MWCO) of 300 kDa, thus proteins or macromolecular complexes above the MWCO would be retained. Control samples that were not separated with the concentrator were saved and analyzed in parallel. Higher amounts of NP protein were detected in the concentrated supernatant (top portion) as compared to the filtrate (bottom portion), which would contain the NP (size 26 kDa) and its homodimers (Fig 1A and 1C). Due to their size, intact RVFV virions would be retained in the concentrated supernatant (top portion). To examine whether infectious virus could be detected within the filtrate i.e. bottom portion, plaque assay analysis was performed on both samples. Comparable infectious titers for both control and top portion samples were observed (1.3E+07 versus 1.2E+07 pfu/ml, Fig 1B). However, roughly 6E+01 pfu/ml of RVFV was detected for the bottom portion. Most of the NP was clearly found in the concentrated top fraction, which also contained the majority of the virions (Fig 1C). However, a detectable amount of NP (approximately 20%) was still present in the filtrate. Calculating the NP band intensity per pfu (Fig 1D) indicated that the filtrate had a greater amount of non-virion associated NP available for potential capture.

### Nanotrap particles can capture RVFV NP

**Fig 2:**
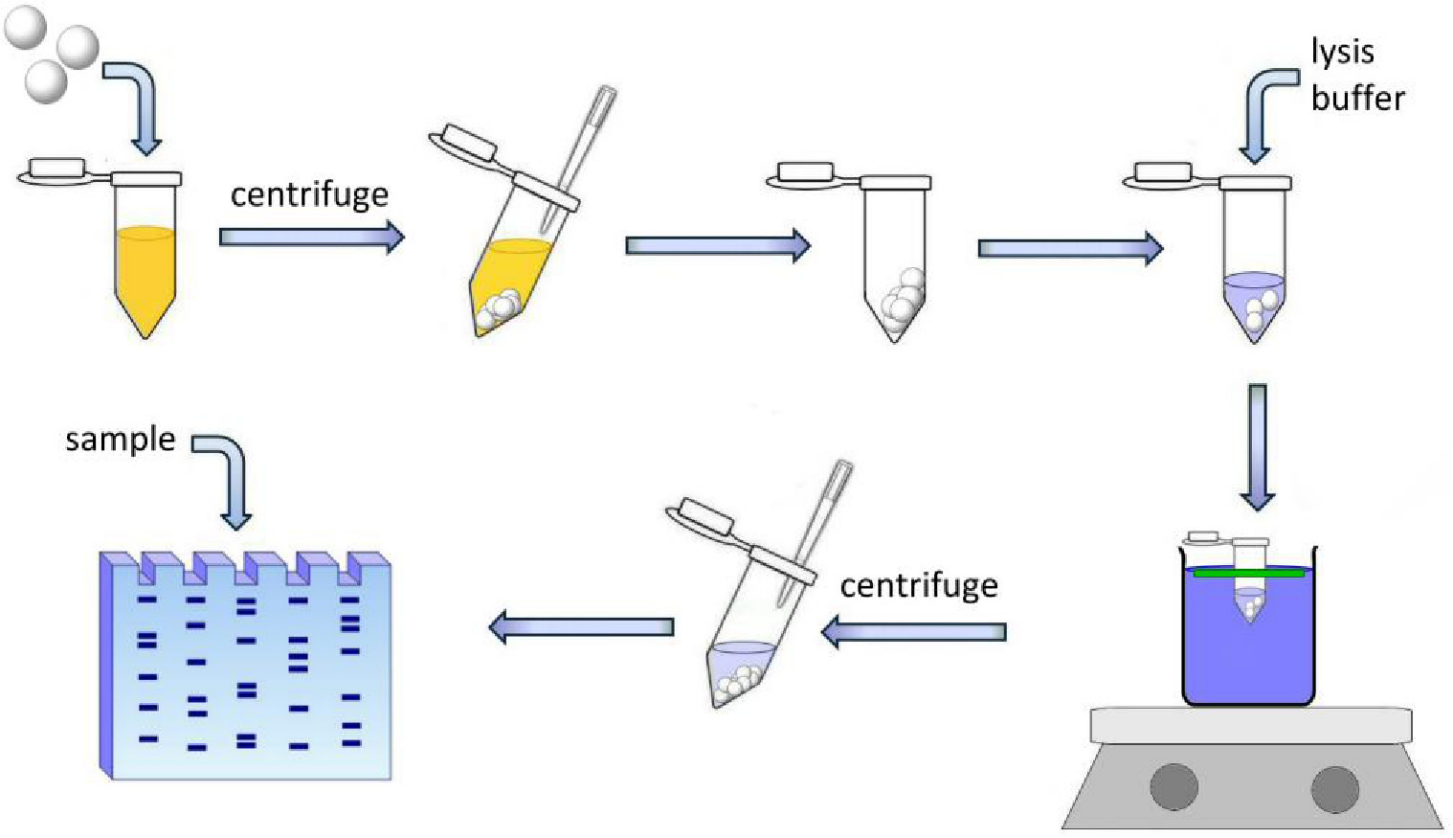
Schematic of antigen capture with Nanotrap particles. Nanotrap particles are incubated with samples for 30 minutes at room temperature, centrifuged, and unbound material is removed. The pellet is resuspended in lysis buffer and boiled for 10 minutes. The sample is then centrifuged and unbound supernatant is loaded onto an SDS PAGE gel.

**Fig 3:**
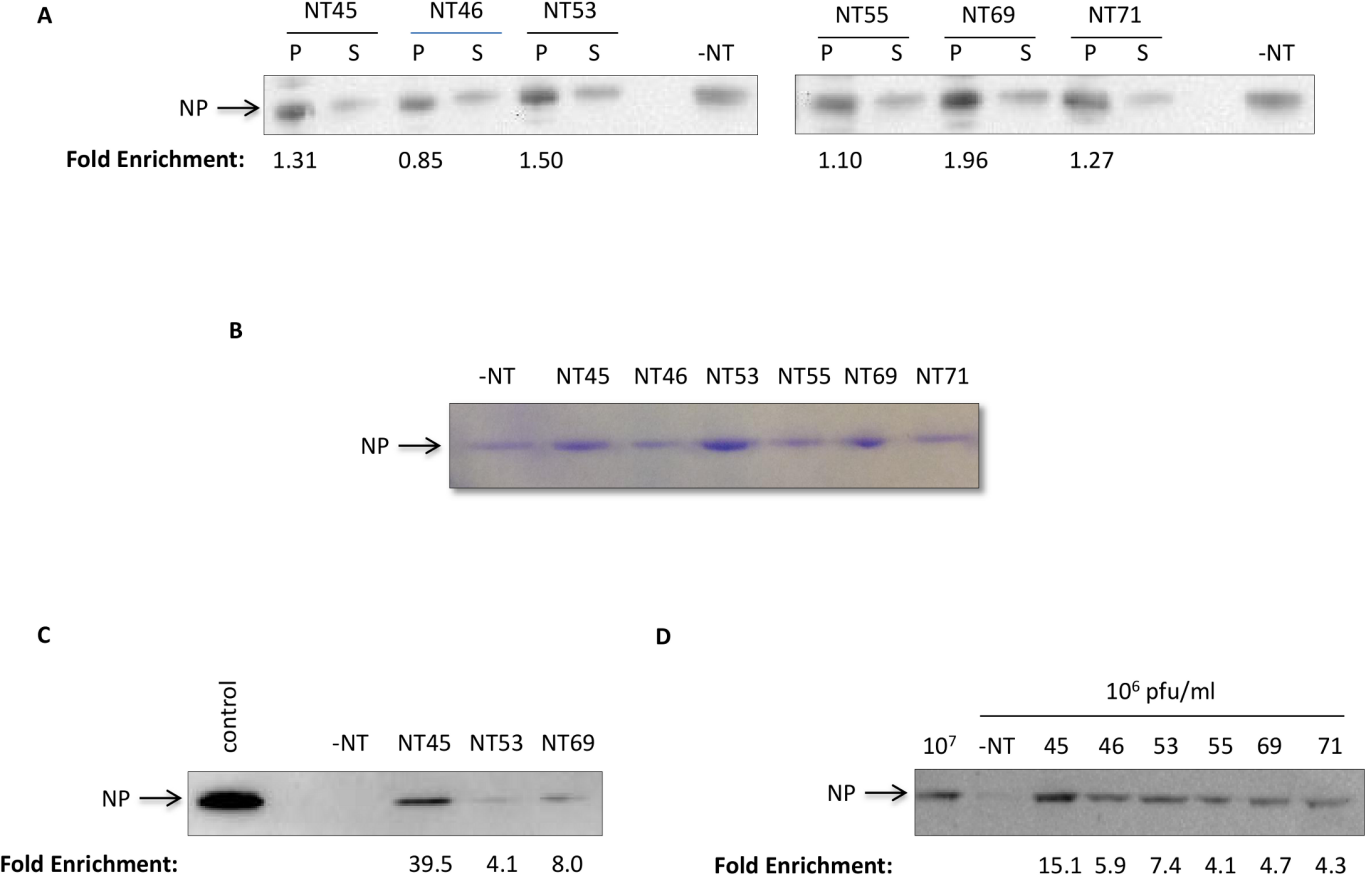
Nanotrap particles can capture RVFV NP. A) Recombinant histidine-tagged NP (His-NP) at a starting concentration of 0.4 mg/ml and a volume of 100 μl was incubated with 75 μl of NT45, NT46, NT53, NT55, NT69, or NT71 for 30 minutes at ambient temperature. After 30 minutes, the (+)NT samples were centrifuged. Both bound (P) and 10uL of unbound (S) material was resuspended in blue lysis buffer and boiled for 10 minutes. The samples were centrifuged at maximum speed and the supernatants were then analyzed for presence of NP protein by western blot using antibodies directed against the histidine tag. No Nanotrap particle samples (-NT) at a 10 μl volume of 0.4 mg/ml His-NP were processed in parallel. B) Purified NP (obtained from BEI Resources) at 2 μg in a volume of 100 μl was incubated with 75 μl of NT45, NT46, NT53, NT55, NT69, or NT71 for 30 minutes at an ambient temperature. A control—NT sample (10 μl volume) was processed in parallel. Samples were processed as describe in panel A. After electrophoresis, NP was visualized by Commassie blue staining. C) His-NP (obtained from Immune Technology) at 1 μg/ml and a volume of one milliliter was incubated with 100 μl of NT45, NT53, and NT69. A control—NT sample (10 μl volume) was processed in parallel. The control sample is 100 μg/ml NP (volume of 10 μl). The samples were processed as in panel A and analyzed by western blotting for NP by using antibodies against NP. D) Viral supernatants at 1E+06 pfu/ml and a volume of 1 ml were incubated with 100 μl NT45, NT46, NT53, NT55, and NT69 for 30 minutes at an ambient temperature. Control—NT samples (10 μl volumes) at 1E+07pfu/ml and 1E+06 pfu/ml were processed in parallel. The samples were processed as in panel A and analyzed by western blot for NP.

Nanotrap particles have been previously used to capture and enrich for various viral proteins and biomarkers. Our subsequent sets of experiments utilized a simple experimental workflow as depicted graphically in Fig 2. To determine whether Nanotrap particles (NT) were capable of capturing and enriching the RVFV NP, particles with various bait chemistries and architectures were screened (Table 1). Purified recombinant His-NP was incubated with seven different Nanotrap particles. Bound (pellet) and unbound (supernatant) sample material were analyzed by western blot (Fig 3A). Our results demonstrated that all Nanotrap particles were capable, to varying efficiencies, of binding His-NP. While 100% capture was not achieved, there was a decrease in the amount of unbound material (S lanes) as compared to the-NT control samples. We confirmed our findings with purified NP (not His-tagged) to ensure that the particles were binding to the NP of the virus and not to the histidine tag (Fig 3B). We obtained similar results demonstrating that NT45, NT53, and NT69 were the best performers with purified recombinant RVFV NP. To demonstrate the binding capacity of the Nanotrap particles with a large sample volume (one milliliter) and low analyte concentration (1μg/ml) we tested the top three performers—NT45, NT53, and NT69 (Fig 3C). These results showed that NT45 was the best performer in enrichment of the protein at a higher volume, with a 39.5-fold increase in NP band density compared to the no NT control. Furthermore, NT45 allowed for the enrichment of the protein at a concentration that is undetectable without Nanotrap particle coupling. Finally, we confirmed NP capture in viral supernatants at a volume of one milliliter. NP was only faintly detectable without Nanotrap particles at 1E+06 pfu/ml. However, there was at least a four-fold increase in NP band density with all six Nanotrap particles screened. Once more, NT45 was the best performer for capturing and enriching NP, with a 15-fold increase in band density levels compared to the no NT control (Fig 3D). Collectively, these results demonstrated that the use of NT45 consistently allowed for the greatest detection of NP.

**Table 1:** Nanotrap Particle Identification Numbers and Bait and Shell Chemistries.

Nanotrap ID	Bait	Shell (y/n)	VSA shell (y/n)
NT45	Reactive red 120 + Reactive yellow 86	No	N/A
NT46	Reactive red 120	No	N/A
NT53	Cibacron blue F3GA	Yes	No
NT55	Acrylic Acid	Yes	N/A
NT69	Cibacron Yellow 3GP	Yes	Yes
NT71	Cibacron Blue F3GA	Yes	Yes
NT104	Reactive Red 120 + Reactive yellow 86	Yes	No

### Nanotrap particles can capture and enrich NP from virally infected cells

**Fig 4:**
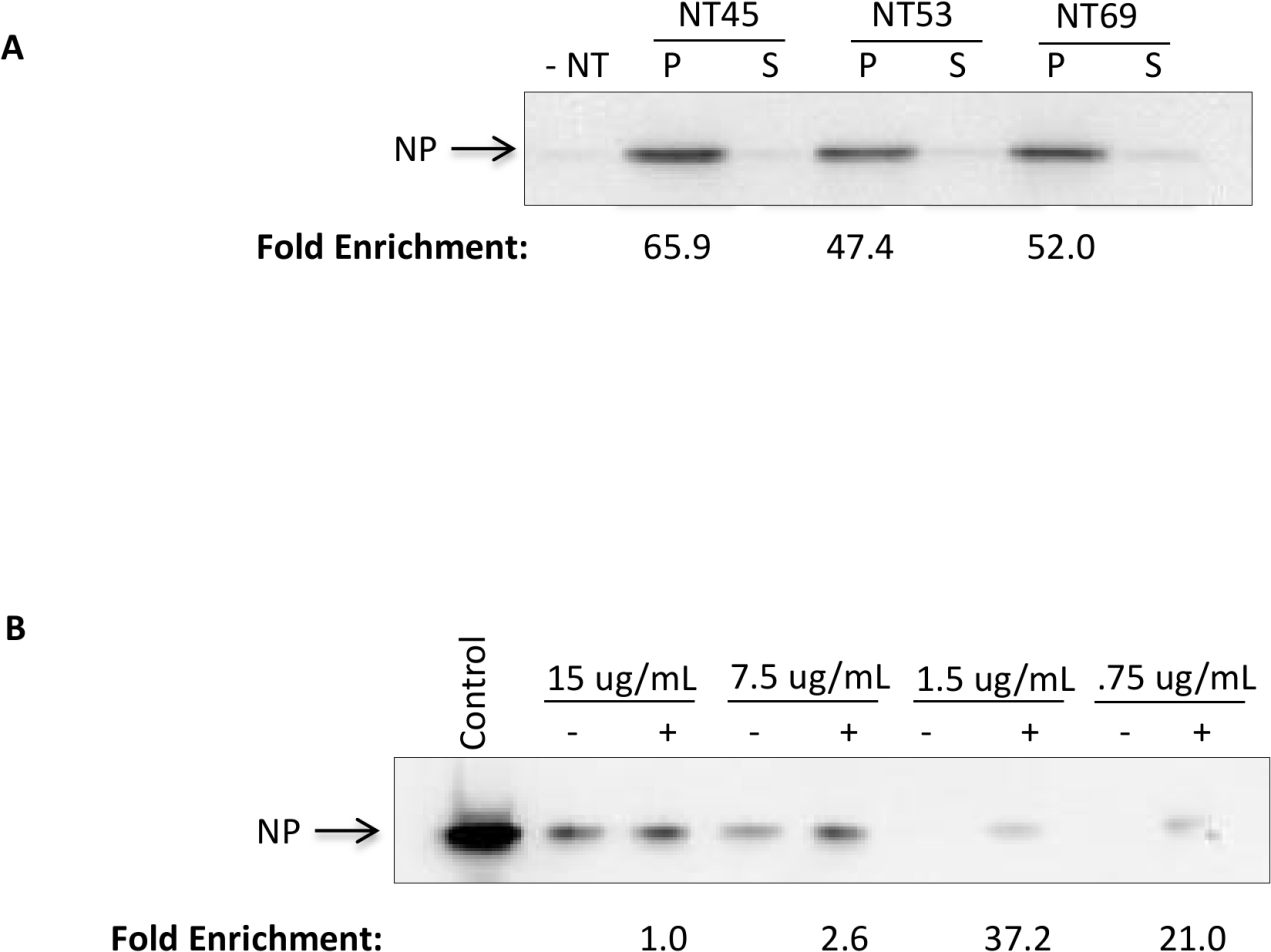
Nanotrap particles can capture and enrich NP from virally infected cells. A) One ml of cytoplasmic extract (CE) at 2.6 μg/ml obtained from RVFV infected Vero lysates were incubated with 100 μl of NT45, NT53, and NT69. No Nanotrap particle sample (-NT) was processed in parallel. After 30 minutes, the (+)NT samples were centrifuged. The unbound material (S) from the spin was saved and 10 μl was processed in parallel. The bound material (P) was resuspended in blue lysis buffer and boiled for 10 minutes. The samples were centrifuged at maximum speed and the supernatants were then loaded onto a NuPage 4–12% Bis-Tris gel. Samples were subsequently analyzed by western blot for NP. B) One ml of CE was serially diluted in 50mM Tris-HCl from 15 μg/ml to 0.75 μg/ml and incubated with 100 μl of NT45 for 30 minutes. No Nanotrap particle samples (-NT) were processed in parallel. The control sample is CE at 770 μg/ml (10 μl volume). The samples were processed as in panel A.

We next wanted to determine the enrichment capability of the Nanotrap particles in infected cell lysates where various host and viral proteins are present. We initially conducted an experiment with our top three Nanotrap particle candidates—NT45, NT53, and NT69. We incubated the three Nanotrap particles with cytoplasmic extracts (CE) obtained from RVFV MP12-infected Vero cells. Our results demonstrated that all three Nanotrap particles were capable of capturing and concentrating NP from a larger volume of 1 ml (Fig 4A). Based on the results from Fig 3C and Fig 4A, we concluded that NT45 yielded the highest increase in band density (66 fold) compared to the no NT sample. We next performed an experiment with four concentrations of CE (15 μg/ml, 7.5 μg/ml, 1.5 μg/ml, and 0.75 μg/ml) to establish a limit of detection with and without NT45. While the NP was detectable without Nanotrap particles at 15 μg/ml and 7.5 μg/ml, the protein was undetectable at the lower protein concentrations. In contrast, the NT45-incubated samples were able to detect the protein down to the lowest concentration tested (Fig 4B), demonstrating a 21-fold increase in band density as compared to the no NT sample tested at the same concentration. Our results indicate that the Nanotrap particles are capable of significantly enriching NP from virally infected cell lysates containing various host and viral proteins.

### Nanotrap particles enhance detection of NP in viral supernatants

**Fig 5:**
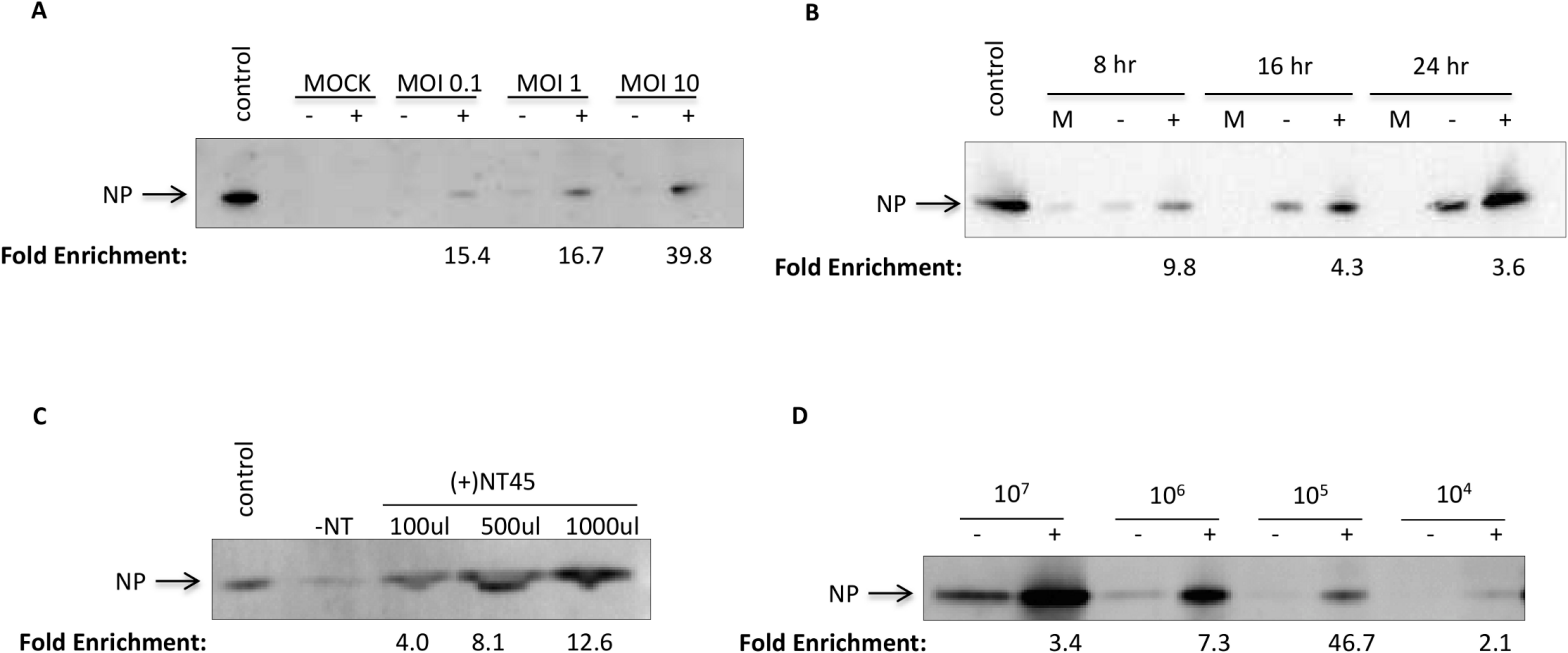
Nanotrap particles can capture and enrich NP from viral supernatants. A) Supernatants from mock infected or MP12 infected (MOI 0.1, 1, or 10) Vero cells were collected at 24 hours post infection. One milliliter of supernatants were incubated with 100 μl of NT45 for 30 minutes. Bound materials were analyzed for presence of NP by western blot using antibodies against NP. No Nanotrap particle (-NT) and mock-infected samples containing no Nanotrap particles (10 μl volumes) were processed in parallel. Control sample is CE at 770 μg/ml (10 μL volume). B) Supernatants from mock infected or MP12 infected (MOI 10) Vero cells were collected at 8, 16, and 24 hours post infection. One milliliter of supernatants were incubated with 100 μl of NT45 for 30 minutes. The samples were processed as in panel A. C) Viral supernatants collected at 48 hours post infection were diluted to a viral titer of 1E+06 pfu/ml. A volume of 100 μl, 500 μl, and 1000 μl were incubated with 100 μl NT45 for 30 minutes. The samples were processed as in panel A. The control sample is viral supernatant at 1E+07 pfu/ml (10 μl volume). D) Viral supernatants (collected 48 hours post infection) at 1E+07 pfu/ml, 1E+06 pfu/ml, 1E+05 pfu/ml, and 1E+04 pfu/ml were incubated at 1 ml volumes with 100 μl of NT45 for 30 minutes. The samples were processed as in panel A.

Since NP is found in both virion and non-virion associated forms, we tested detection of NP in viral supernatants of Vero cells infected with RVFV MP12. Utilizing various MOIs (0.1, 1, and 10) and timepoints (8, 16, and 24 hr) we found that the detection of NP with the addition of NT45 increased as the MOI increased, leading to a 39.8-fold enrichment at an MOI of 10 as compared to the no NT sample (Fig 5A). Furthermore, detection of NP with and without NT45 increased from 8 to 24 hr post-infection (Fig 5B). While still detectable at 8 hours without the addition of Nanotrap particles, there was a near 10-fold increase in NP band density with the addition of NT45. We also compared different sample volumes with the addition of NT45 to demonstrate the enrichment capabilities of the Nanotrap particles (Fig 5C). While the NP was virtually undetectable in the control samples containing no Nanotrap particles, a clear band was detected at 100 μl of sample with band intensity increasing as the volume of sample increased, with a 12.6-fold increase in band density at the 1 ml sample volume compared to the no NT sample. Lastly, we wanted to determine if we could increase the limit of detection with the Nanotrap particles. We incubated viral supernatants at various viral concentrations from 1E+07 to 1E+04 pfu/ml with NT45 (Fig 5D). Comparable to what was previously seen in Fig 3D, the no Nanotrap samples were barely detectable at 1E+06 pfu/ml. In contrast, there was a 7.3-fold increase of NP band density with the addition of NT45 at 1E+06 pfu/ml, 46.7-fold increase at 1E+05 pfu/ml and a 2.1-fold increase at 1E+04 pfu/ml. It is important to note that NT45 may be binding to virion and non-virion associated NP in these experiments. These results show that NT45 enhanced the detection of all forms of NP found in viral supernatants at various timepoints, MOIs, viral concentrations, and volumes.

### Nanotrap particles enhance detection of NP in animal sera

**Fig 6:**
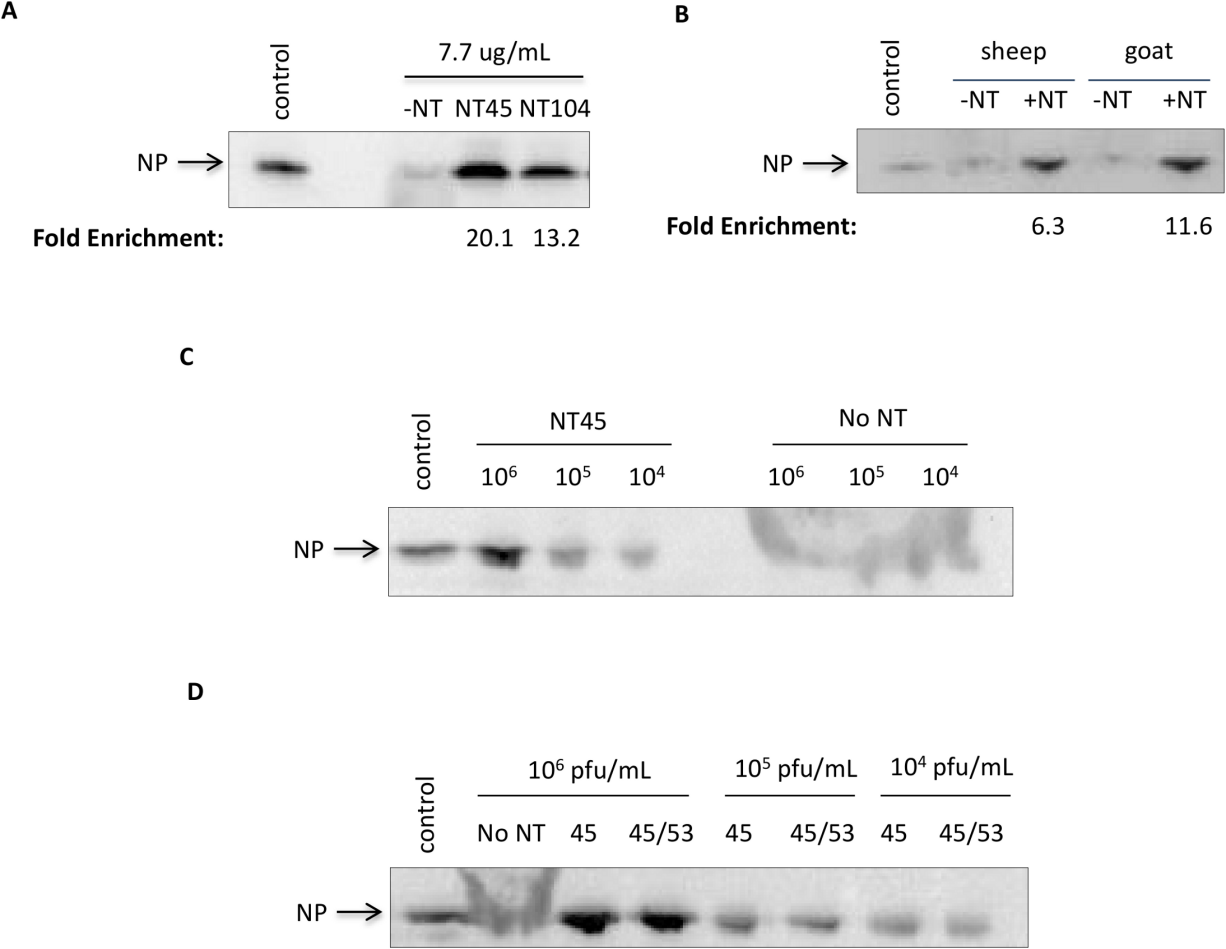
Nanotrap particles can capture and enrich NP found in various animal sera. A) Cytoplasmic extracts from RVFV infected cells were diluted to 7.7 μg/ml in 100% sheep serum. One milliliter of sample was incubated with 100 μl of NT45 or NT104 for 30 minutes. No Nanotrap particle (-NT), control (CE at 77.7 μg/ml diluted in 50mM Tris-HCl), and mock-infected samples at 10 μl volumes were processed in parallel. The (+)NT samples were then centrifuged and washed once with 0.25M sodium thiocyanate and twice with diH_2_O. The samples were analyzed by western blotting for NP using antibodies against NP. B) Cytoplasmic extracts from RVFV infected cells were diluted to 7.7 μg/ml in 100% sheep, goat, or donkey sera. One milliliter of sample was incubated with 100 μl of NT45 for 30 minutes. No Nanotrap particle (-NT) and control (CE at 7.7 μg/ml diluted in 50mM Tris-HCl) samples at 10 μl volumes were processed in parallel. The samples were processed as in panel A. C) Viral supernatants were diluted in sheep serum for final viral titers of 1E+06 pfu/ml, 1E+05 pfu/ml, and 1E+04 pfu/ml. One milliliter of the sample was incubated with 100 μl of NT45 for 30 minutes. Control sample is viral supernatant at 1E+07 pfu/ml (10 μl volume). The samples were processed as in panel A. D) Viral supernatants were diluted in sheep serum for final viral titers of 1E+06 pfu/ml, 1E+05 pfu/ml, and 1E+04 pfu/ml. One milliliter of the sample was incubated with 100 μl of NT45 or 100 μl of equal parts NT45 and NT53 for 30 minutes. Control sample is viral supernatant at 1E+07 pfu/ml (10 μl volume). The samples were processed as described in panel A.

We next wanted to mimic a native infection scenario using animal sera (sheep and goat) to demonstrate the enrichment capabilities of the Nanotrap particles in these complex solutions containing various host proteins (such as albumin). NT45, our best performing candidate, is a Nanotrap that contains reactive red 120 and reactive yellow 86 chemical dye baits without a surrounding outer shell. The outer shell serves to further exclude high abundant, high molecular weight analytes that may compete with lower molecular weight proteins. To determine if the inclusion of an outer shell would allow for better enrichment of NP in more complex media (e.g. animal sera), NT104 was created, which is NT45 containing an outer shell. We initially started with 100% sheep serum spiked with cytoplasmic extracts from RVFV infected cells incubated with NT45 and NT104. We hypothesized that the outer shell of NT104 would exclude the high abundant host proteins and increase NP detection. However, we found a striking enrichment of the NP with both NT45 and NT104, whereas the control (no NT) sample was only slightly detectable (Fig 6A). Surprisingly, we found that NT45, not NT104, was the best candidate in serum capture, resulting in a 20-fold increase in band density for NT45 and a 13-fold increase in band density for NT104 compared to the no NT sample. These results suggest that the outer shell of NT104 may interfere with capture of the dimeric form of the NP. We therefore utilized NT45 in the capture of NP in both sheep and goat sera. Similarly NP was greatly enriched in goat serum, with a 6- and 12-fold increase in band density for sheep serum and goat serum, respectively (Fig 6B). Next, we wanted to determine the limit of detection of RVFV in sheep serum. In order to capture all forms of NP (monomeric and dimeric), we incubated a combination of NT45 and NT104 particles with 1 ml samples of sheep serum spiked with RVFV MP12 supernatant at various titers. While NP was not detectable at any viral titers tested without Nanotrap particles, the NP was detectable down to 1E+04 pfu/ml after NT45/104 incubation (Fig 6C). Lastly, we wanted to demonstrate whether a combination of Nanotrap particles would be best for NP enrichment. Previously we have shown that NT53, which contains a cibacron blue bait and an outer shell, was the best Nanotrap particle for capture of RVFV virions, as demonstrated with both plaque assays and qRT PCR [11]. We therefore incubated our sample with equal parts NT45 and NT53 and compared capture to NT45 alone at various viral concentrations. Our results showed that there was not a significant increase in NP detection with the combination of Nanotrap particles (Fig 6D). This result is not entirely unexpected as NT45 was also capable of capturing RVFV virions, albeit at a lower efficiency than NT53 [11]. These results demonstrate that the Nanotrap particles are able to capture and enrich for both virion and non-virion associated NP, allowing for the enhanced detection of NP without interference of other high abundant proteins that are found in clinically relevant matrices.

### Nanotrap particles can protect NP from degradation at increased temperatures and times

**Fig 7:**
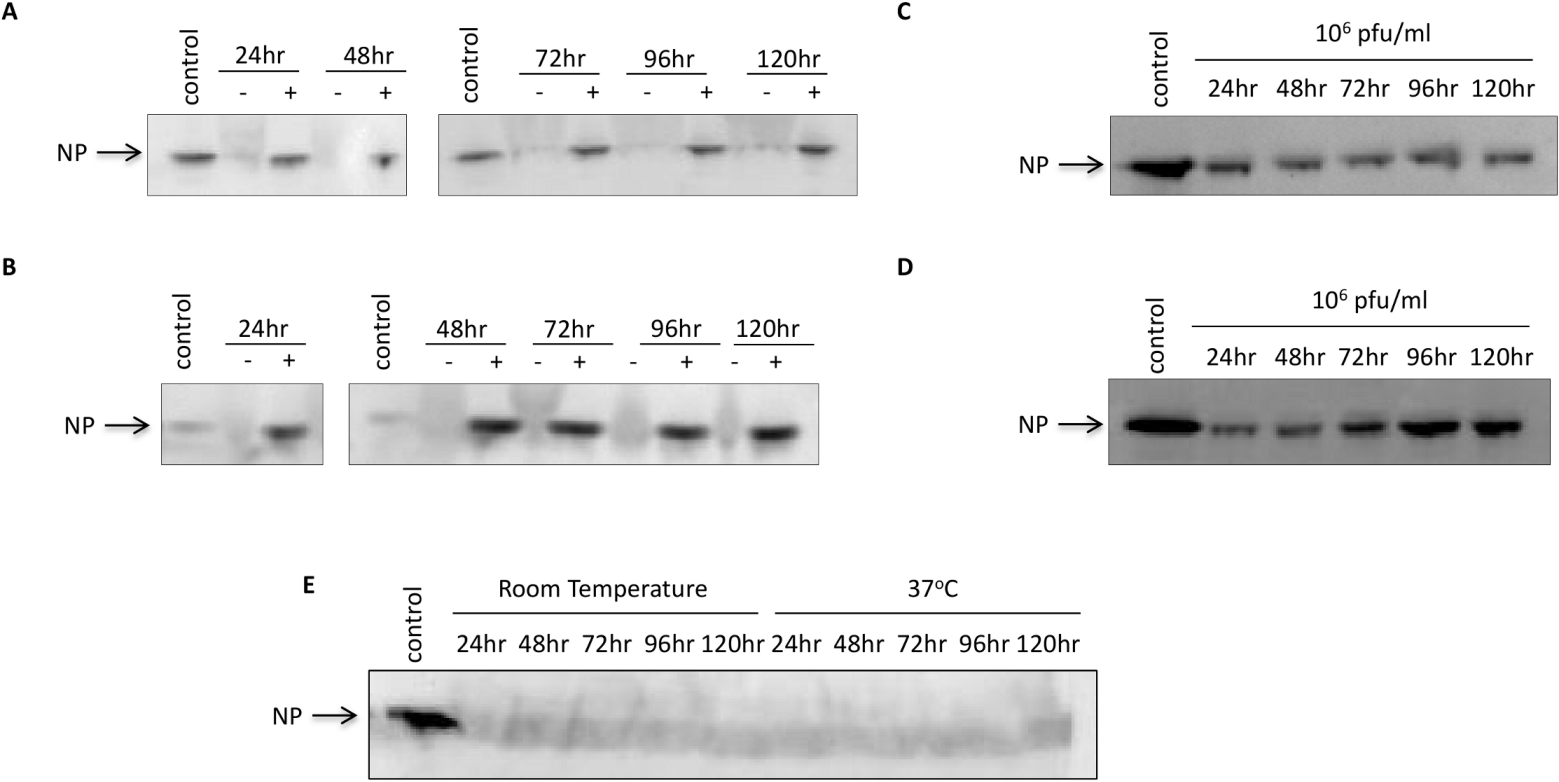
Nanotrap particles can protect NP from degradation at increased temperatures and times. A and B) Cytoplasmic extracts from RVFV infected cells were diluted in 100% sheep serum for a final concentration of 7.7 μg/ml. C and D) Viral supernatant was diluted in 100% goat serum for a final titer concentration of 1E+06 pfu/ml. One milliliter of sample was incubated with NT45 for 24 to 120 hours at either ambient temperature (A and C) or at 37^°^C (B and D). No Nanotrap particle (-NT) and control cytoplasmic extract (CE) samples at 7.7μg/ml or 0.77 μg/ml (B only) at 10 μl volumes were processed in parallel. Viral supernatants without Nanotrap particles containing samples were analyzed on a separate gel (E). After the incubation time, the (+)NT samples were centrifuged and washed once with 0.25M sodium thiocyanate and twice with diH_2_O. Presence of NP was analyzed by western blot using antibodies against NP.

A critical problem in viral diagnostics is the preservation of samples during transport. Most viral proteins are degraded in the presence of naturally occurring proteases found in sera. Furthermore, exposure to heat increases viral protein degradation. Our next set of experiments aimed to determine if Nanotrap particles were capable of protecting NP from degradation after prolonged storage (up to 120 hours) and at an elevated temperature (37°C). We examined both NP in cytoplasmic extracts as well as in viral supernatants spiked in sheep or goat sera. We first tested NP stability at ambient temperatures over a long incubation period. The samples incubated with NT45 remained stable from 24 to 120 hours for both cytoplasmic extracts and viral supernatants (Fig 7A and 7C, respectively). In the absence of Nanotrap particles, NP was virtually undetectable at this dilution (as seen in Fig 7E for viral supernatants). We next wanted to demonstrate that the Nanotrap particles can protect NP at elevated temperatures. The Nanotrap particles are temperature-sensitive, decreasing in size with increased temperature [17]. We therefore hypothesized that in the presence of elevated heat, the Nanotrap particles would decrease in size and further restrict NP inside. Once again, NP in the absence of the Nanotrap particles was virtually undetectable starting at 24 hours (as seen in Fig 7E for viral supernatants). In contrast, NP from the NT45 samples was strongly detected at all timepoints from 24 to 120 hours for both sample sets (Fig 7B and 7D). Furthermore, over time there was no NP degradation in the presence of NT45. Collectively, these results demonstrate that Nanotrap particles are capable of protecting NP (and perhaps the virion itself) from degradation during prolonged storage at elevated temperatures.

## Discussion

Nanotrap particles have traditionally been used for the capture and enrichment of proteins [7–10,18]. Several papers have shown that these hydrogel particles enrich numerous proteins such as insulin, myoglobin, and PDGF [7,8]. A 2011 paper by Douglas *et al*. demonstrated that the Nanotrap particles can dramatically concentrate Lyme disease antigens in urine and enhance detection at previously undetectable concentrations [10]. The Nanotrap particles can also be used for the capture of intact virions. We previously demonstrated the capture and enrichment of RVFV virions and other viral pathogens with several Nanotrap particles [11]. Importantly, we showed that while the virus can be inactivated with heat or detergent after Nanotrap particle incubation, the nucleic acid of the virus is still detectable with qRT-PCR methodology. We have expanded upon these previous findings and shown here that the Nanotrap particles can be utilized as a sample preparation tool to concentrate NP that is commonly used for RVFV diagnostics. Specifically, RVFV NP can be concentrated from a large starting volume of sample into a small volume from clinically relevant matrices, as demonstrated by western blot.

Our first goal was to determine the compatibility of the Nanotrap particles with the analyte of interest. From our initial Nanotrap particle screening we identified NT45, NT53, and NT69 as our top three candidates, all of which are triazine-derived textile dyes that have commonly been used for protein purification via affinity chromatography [19]. The structure of the dyes mimic the structure of substrates that bind to the active sites of different proteins and therefore are able to bind a wide variety of proteins. Dye affinities are determined by the structure of the dye and how well it can bind to the active site of the proteins in question. NT45 is one core particle that is coupled to two different dyes; it is a mixture of reactive red and reactive yellow dyes, whereas NT53 contains cibacron blue baits. Cibacron blue has been described as a “universal pseudoaffinity ligand” that nonspecifically binds analytes through ionic and/or hydrophobic interactions due to its aromatic (nonpolar) and sulfonate (ionic) groups [20]. While the exact mechanism in which the Nanotrap particles bind to the analyte is unknown, we hypothesize that the baits of the three Nanotrap particles are somehow attracted to the hexameric structure of the NP in an ionic manner [20,21]. Specifically, the Nanotrap particles may be binding to the positively charged patch of the NP hexamer that constitutes the RNA binding site [20,21].

A common problem in diagnostics is the degradation of proteins soon after sample collection. This is due to several factors including both endogenous and exogenous proteases, as well as temperature and pH changes. It is therefore recommended that samples be placed in temperatures of 4°C or lower for short-term transport and -80°C for long-term storage [22]. However, these temperature settings are not always possible in a field environment. Nanotrap particles have previously been found to protect analytes from proteolytic degradation by trapping the protein of interest within the particles while simultaneously excluding proteases such as trypsin [8,9]. Furthermore, the hydrogel particles shrink at elevated temperatures above 34°C [23], further trapping the analytes within the particles. We have expanded upon these findings by demonstrating that the Nanotrap particles can not only capture and enrich RVFV NP in the presence of abundant resident proteins that are found in serum, but also protect the viral antigen from degradation for at least 120 hours at both ambient and elevated temperatures. This sample preparation technology will allow for sufficient collection and transfer of clinical samples from a field or hospital setting to a laboratory for subsequent testing.

Significant enhancement in terms of sample enrichment and subsequent protection of RVFV NP with Nanotrap particles, as detected by western blot, was observed. This NT capture method could be coupled to other downstream assays. Sensitivity issues are a common problem during ELISA diagnostics as viral titers often fall below the threshold of detection. While viral titers in RVFV-infected individuals can be very high, titers start to decrease after a short period of time. For antigen (Ag)-capture ELISA, samples must be collected during a small window of time (ranging from 2 to 6 days) or false-negative results may occur [5,24]. Our western blot results suggest a 100-fold increase in NP detection after Nanotrap particle incubation. Specifically, our limit of detection experiments demonstrated the enhanced detection of NP after Nanotrap particle incubation at low viral titers that would have normally rendered a false-negative result. We anticipate similar results with Ag-capture ELISAs. Our future goals are to utilize the Nanotrap particles to exclude the high-abundant molecules while concentrating NP in high-volume samples (up to 10 ml). Our aim is to increase the sensitivity range for Ag-capture ELISAs to be comparable or exceeding that of current PCR methodologies. Some buffers that efficiently elute proteins out of the Nanotrap particles can interfere with antigen-antibody capture for ELISAs. Therefore, our goal is to identify an elution method that efficiently elutes our sample off of the particles and is compatible with ELISAs. Alternatively, new generations of Nanotrap particles that are "degradable" may provide an effective solution to coupling the particles with ELISAs.

While the promiscuity of these particles allows for more than one protein type to be captured, our results demonstrated that this feature does not interfere with the Nanotrap particle's enrichment capability. This feature is favorable when the cause of infection is unknown as several viral infections can result in the same symptoms. An example of this is with RVFV and other arboviruses. After a bite from an infected mosquito, symptoms for these arthropod-borne infections are similar, and range from mild (slight fever, headache, body aches) to severe (high fever, tremors, convulsions). The Nanotrap particles’ ability to concentrate various antigens and/or nucleic acids allows for analysis using multiplex assays (e.g. multiplex PCR, Luminex) containing a large panel of primers or antibodies targeted against pathogens that induce these similar symptoms.

The Nanotrap particles also serve as an important tool for host biomarker discovery. As previously mentioned, the promiscuity of the Nanotrap particles allows for the enrichment of both host and viral antigens from a single sample. Since host protein biomarkers are typically of low abundance and size, they are easily missed in favor of large, high-abundant molecules that make up the majority of the circulating proteins, with albumin itself accounting for 55% of the proteins found in plasma samples [25]. This is a common problem with mass spectrometry, which requires a very small sample size and starting mass in the range of 0.1–10μg per protein [26]. The size sieving property of the Nanotrap particles excludes large biomolecules while concentrating small, low-abundant biomarkers. A number of recent papers have demonstrated the enhanced detection of several biomarkers with these particles. Longo *et al*. demonstrated the detection of the platelet-derived growth factor protein at previously undetectable levels [17]. Another study demonstrated the sequestration and concentration of the host cytokines interleukin-6 (IL6) and interleukin-8 (IL8) with Nanotrap particles. Importantly, these proteins are protected from enzymatic degradation [18]. These findings can be expanded to RVFV biomarker discovery. A recent paper published by Narayanan *et al*. successfully utilized Nanotrap particles to compare IL8 concentrations in mock-infected and MP12-infected samples [27]. Collectively, these findings suggest that Nanotrap particles can be utilized to help analyze the alterations of host genes after RVFV infection in a more sensitive manner.

We have demonstrated that the Nanotrap particles are a reliable and quick sample preparation technology that allows for the enrichment from a large sample volume without the interference of highly abundant serum proteins or protein degradation over an extended time and elevated temperatures. Concentrated samples can be efficiently eluted with only a small volume of eluent and further processed with assays such as SDS PAGE for additional analysis. Our future projects will investigate the feasibility of extending this concept to other downstream methodologies, which will include ELISA, mass spectrometry, and Luminex assays. Lastly, we hope to couple the Nanotrap particles to point-of-care diagnostic devices that can rapidly (<30 minutes) detect various pathogens in a field or clinical setting.
